# Gaze direction influences grasping actions towards unseen, haptically explored, objects

**DOI:** 10.1038/s41598-020-72554-x

**Published:** 2020-09-25

**Authors:** Martina Pirruccio, Simona Monaco, Chiara Della Libera, Luigi Cattaneo

**Affiliations:** 1 Department of Neurosciences, Biomedicine and Movement Sciences, University of Verona, 37134 Verona, Italy; 2 Center for Mind/Brain Sciences, University of Trento, Via delle Regole, 101, 38122 Trento, Italy

**Keywords:** Cognitive neuroscience, Sensorimotor processing, Somatosensory system, Visual system

## Abstract

Haptic exploration produces mental object representations that can be memorized for subsequent object-directed behaviour. Storage of haptically-acquired object images (HOIs), engages, besides canonical somatosensory areas, the early visual cortex (EVC). Clear evidence for a causal contribution of EVC to HOI representation is still lacking. The use of visual information by the grasping system undergoes necessarily a frame of reference shift by integrating eye-position. We hypothesize that if the motor system uses HOIs stored in a retinotopic coding in the visual cortex, then its use is likely to depend at least in part on eye position. We measured the kinematics of 4 fingers in the right hand of 15 healthy participants during the task of grasping different unseen objects behind an opaque panel, that had been previously explored haptically. The participants never saw the object and operated exclusively based on haptic information. The position of the object was fixed, in front of the participant, but the subject’s gaze varied from trial to trial between 3 possible positions, towards the unseen object or away from it, on either side. Results showed that the middle and little fingers’ kinematics during reaching for the unseen object changed significantly according to gaze position. In a control experiment we showed that intransitive hand movements were not modulated by gaze direction. Manipulating eye-position produces small but significant configuration errors, (behavioural errors due to shifts in frame of reference) possibly related to an eye-centered frame of reference, despite the absence of visual information, indicating sharing of resources between the haptic and the visual/oculomotor system to delayed haptic grasping.

## Introduction

### Representation of haptically-acquired object shapes in the central nervous system

As with most primates, our daily object-directed actions rely mainly on vision. However, when vision is not available, we need to use information from other senses in order to interact with our surroundings. When it comes to hand-object interactions, the sense that allows us to accurately extract information about the characteristics of objects within reach, such as their size, shape and location, is touch. The canonical model of haptic information processing for object manipulation depicts a circuit involving the ventral caudal nucleus of the thalamus, the anterior parietal cortex and two parallel streams through the secondary somatosensory cortex and the insula on one side and through the superior parietal lobule on the other^1^. Such robust model has been repeatedly confirmed in non-human and human primates on the basis of physiological, imaging and neuropsychological data^2–5^. In the recent years, however, a growing body of evidence points to a role of the early visual cortex (EVC) in processing haptic information. Activation of striate and extrastriate visual regions during tactile identification of objects has been observed in sighted individuals, even when visual information about the object was not available, and regardless of whether the haptically-explored shapes were familiar or not^2,6–11^. Moreover, the EVC also shows reactivation during grasping actions in the dark towards an object that has been haptically explored seconds earlier^12^. In addition, evidence for the potential existence of an access pathway of tactile information to the EVC is provided by studies in blind individuals. In the congenitally blind, the EVC is activated during Braille reading or tactile perception^13–17^ thanks to neuroplasticity driven by sensory deprivation. Overall, these results show evidence that not only the EVC is involved in haptic exploration of objects, but it is also reactivated during subsequent grasping actions in the dark, suggesting that grasp-relevant properties about the object might be recruited from the EVC at the time of action even in absence of online visual information.

### Frames of reference in vision for action

A crucial question in sensorimotor processes is related to how the brain transforms sensory information in different frames of reference, to be used for movements. A target’s shape in the EVC is coded in retinal coordinates. This information must be integrated with that on eye position to build a head-centred image and this is further integrated with head-, body- and hand-position in space to obtain an ultimate image in an egocentric frame of reference that is functional to hand-object interactions^18^. Visual information necessarily undergoes such process of subtraction of gaze position to reach the premotor cortex. The more distal cortical nodes where retinotopic and eye-centred images are found along the dorsal visual stream are seemingly the lateral intraparietal area (LIP) and the frontal (FEF) and supplementary eye fields (SEF)^19–21^. The premotor cortex, is generally considered to be “gaze-independent”, at least for representations of objects for grasping^22^, though recent works indicated the presence of retinotopic coding in the monkey’s premotor sector^23,24^. Further, neuroimaging evidence in humans shows that gaze direction modulates the activity in the parieto-frontal network, which is known to be crucial for motor behaviour, during reaching movements towards visual targets^25–27^. Such an influence appears to be reflected on behavioural data as well, so that eccentric gaze directions induce larger grip aperture, consisting of the distance between the index finger and the thumb, during a reaching movement towards a visual target, presumably in order to facilitate contact with the target^28,29^. Grip aperture is used as an index of hand shaping in motor behaviour, and it correlates with target size^30^.

Summing up, haptic information of objects, acquired in a hand-centred frame of reference is stored in “visual” buffer in the EVC and information in the EVC needs to integrate gaze position to be used in grasping movements. Such a “visual” storage, albeit temporary, could be useful for the genesis of movement towards objects, after appropriate integration of object information in a more complex spatial map that includes other coordinate systems, such as hand-, body- or head-centred ones.

### Is haptic information remapped in eye-centred coordinates?

These concepts raise the question about whether gaze direction affects grasping movements towards haptically explored objects, like it does for visually explored objects. Yet, while most behavioural studies have focused on how eye-position affects grasping movements towards visually explored targets, the effects of gaze direction on movements towards haptically-explored targets has not been investigated yet, and this is the specific experimental hypothesis that we are addressing in the current work. In other words, the question is, if the perceptual representation of a target is stored in a visual sketchpad, then is this mapped in eye-centred coordinates irrespective of the sensory modality initially used to explore the target? Specifically, is the memory-based perceptual representation of a target mapped in eye-centred coordinates even if the target is explored or located with senses other than vision, like proprioceptive or haptically-explored targets? Neuroimaging evidence suggests that in sighted individuals, parietal areas code targets in gaze-centred coordinates for grasping actions, irrespective of whether the target to be grasped is visual or proprioceptive, i.e. a 3D real cube or one’s own hand^31^. In addition, in blind individuals spatial updating of proprioceptive target location for reaching movements depends on gaze direction^32^. Therefore, we reasoned that if the perceptual representation of a target is encoded in eye-centred coordinates regardless of the modality in which the target has been perceived (or located), it is possible that gaze direction affects grip aperture during grasping actions towards occluded objects that have been haptically explored. On the other hand, should gaze direction have no effect on delayed grasping actions towards haptically explored objects, which might be instead represented in hand-centered reference frames, shifts in gaze position relative to a constant object location (and constant hand starting-position) should have no influence on subsequent movements. Grip aperture is the measure of the distance between the fingertips and has a prominent role in the study of sensorimotor transformations for grasping. When reaching for an object under visual guidance, the hand is pre-shaped to accommodate the object’s geometry, and maximal grip aperture during reach is a measure that is highly informative of the capacity of the brain to build a visual representation of the object for acting upon it^33^.

To investigate whether grasping movements towards tactile stimuli are influenced by gaze direction, we conducted a behavioural experiment in which participants haptically explored a cylindrical object of three possible sizes aligned with body midline, and subsequently grasped it. The object was occluded from the participant’s view throughout the duration of the experiment. We manipulated gaze direction such that participants fixated towards the object or away from it. We hypothesized that if an haptically explored object is mapped in eye-centred coordinates, then grip aperture would change when participants grasp the object while fixating peripheral as opposed to central locations. This would occur for two possible reasons: in the peripheral visual field, uncertainty about the properties (i.e. size) of the object to be grasped would increase and the remembered location of the object would conceivably also occupy different eccentricities in retinotopic space and therefore be warped by the viewpoint.

In this way we aim to highlight “configuration errors”^34^, i.e. behavioural errors due to shifts of reference frames. These are generally evoked by changes in viewpoint or body part position and are widely employed in the cognitive neurosciences (as for example in Bisiach and Luzzatti^35^). The finding of configuration errors is an indicator that a given behavioural function is actually based on a given reference frame. While previous studies have investigated the effect of gaze direction on maximum grip aperture of the thumb and index finger, as in a precision grip^36^, we examined the influence of gaze on all fingers involved in a precision whole-hand grasp in order to provide a comprehensive view of the kinematics of all fingers that participate in hand actions. In addition, we performed a control experiment to test whether gaze direction affects non-goal-directed fingers movements, i.e. in the absence of an object or its mental representation.

## Materials and methods

### Participants

Fifteen participants (8 females) with age ranging from 20 to 32 (mean age ± SD: 24.9 ± 4.5) took part in the main experiment. A control experiment was carried out with another set of 15 participants (12 females) with age ranging from 20 to 33 (mean age ± SD: 27 ± 3.9). All subjects were right-handed, and had no neurological, psychiatric or other medical condition. All volunteers gave their written informed consent before the experimental session. The experimental protocol was approved by the ethic committee of University of Verona and was carried out in accordance with the ethical standards of the 2013 Declaration of Helsinki.

### Apparatus

**Figure 1:**
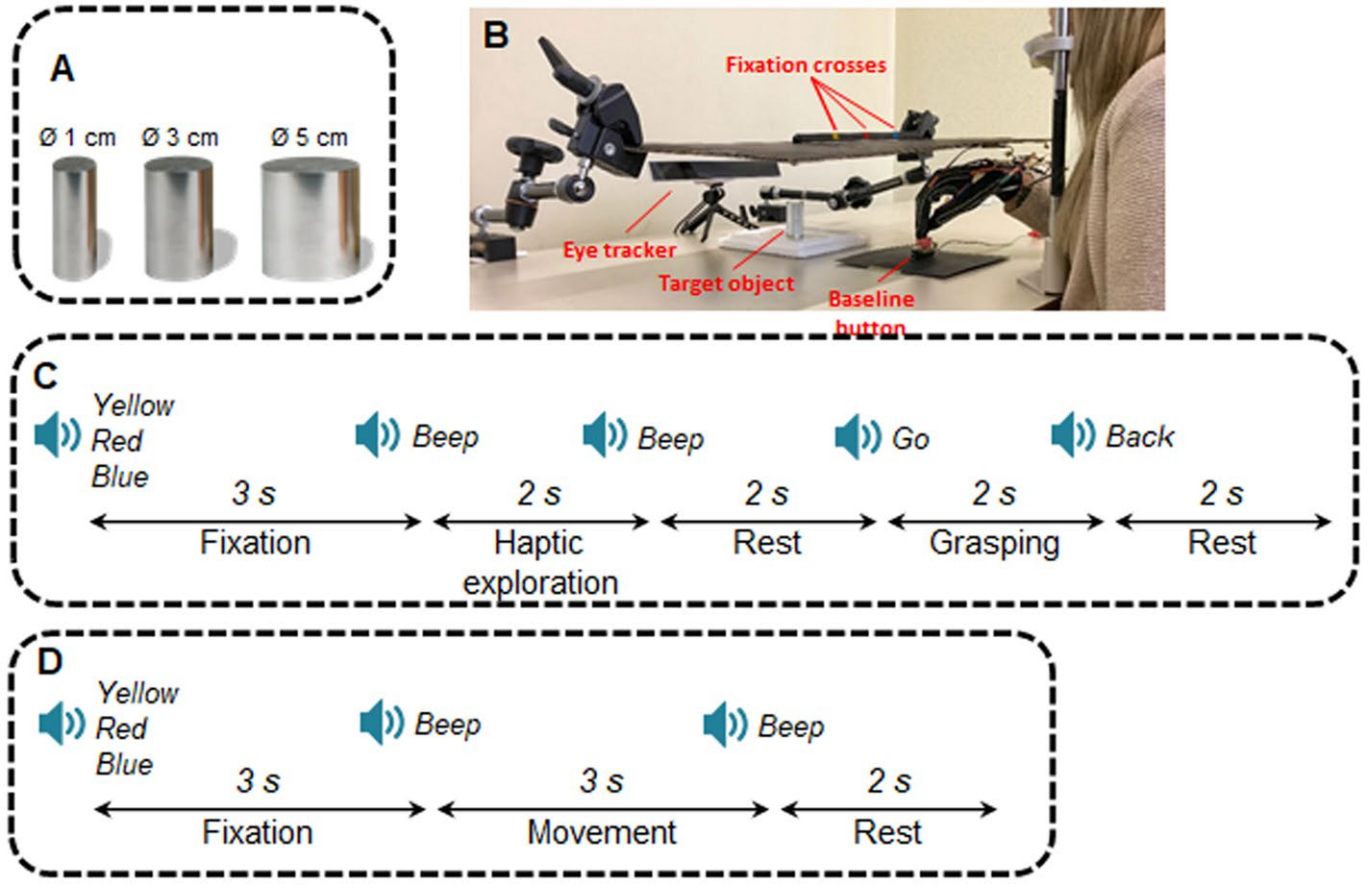
Experimental setup and paradigm. (**A**) Objects used in the main experiment. The three cylindrical objects had a height of 7 cm and diameter of 1, 3 and 5 cm. (**B**) Experimental setup. (**C**) Timing of trials in the main experiment. (**D**) Timing of trials in the control experiment. The Figure was processed by means of the Microsoft PowerPoint software and the GIMP (Gnu Image Manipulation Program) software.

Participants sat comfortably in front of a table where one of three differently sized metallic cylindrical objects (height of 4 cm and radiuses of 1, 3 or 5 cm) was placed by the experimenter in between trials (Fig. 1A). The object was hidden from the participant’s view by means of a black panel placed ~ 17 cm above a table (Fig. 1B). During the experiment participants fixated one of three fixation crosses of different colours (blue on the left, red in the middle, yellow on the right) on the black panel. The central fixation cross and the occluded object were aligned with the participant’s body midline, while the lateral fixation marks were placed 8.5 cm to the left and to the right of the central cross. The distance of the participants’ eyes from the central cross was 30.9 ± 2.5 cm; thus, the eccentric gaze directions were at 15.6 ± 1.3° of visual angles from the central fixation. For each participant, the panel was installed using a pointer, so that the subject’s gaze trajectory towards the central fixation cross coincided with the location of the cylindrical object hid below the panel. In other words, when looking at the central fixation participants would have been looking directly at the object if the panel had been removed. The panel was supported by two later mechanical arms; a spirit level was used to ensure that the surface of the panel was perfectly level on the horizontal plane. The subjects’ head was fixed on a chin rest (mean height ± SD: 28.8 ± 2.9) to avoid head movements while looking at the lateral fixation crosses during the experiment. A button was placed on the table between the chin rest and the object indicating the baseline position, during which participant rested the right hand on the button with all fingers pointing down on it (Fig. 1B).

### Procedure

Participants sat in front of a black panel with three fixation crosses (left, central and right). A button was placed in front of the subjects and served as the baseline position. Participants pressed the button with the fingers closed before and after each trial. In the main experiment, the panel hid one of the three objects. For each experimental session, the panel was set with a spirit level at a suitable distance for each participant so that gaze direction towards the central fixation cross coincided with the location of the object hid below the panel. The Gazepoint GP3 eye-tracker allowed us to control for participants’ fixation and ensure that they could reliably fixate the instructed fixation point for the duration of each trial. The same setup was used for the control experiment, with the only exception that no object was used.

Each trial began with a recorded voice saying the colour of one of the three fixation crosses (“blue” for left, “red” for centre, “yellow” for right), and prompting the participants to fixate the cued cross for the whole duration of the trial. After 3 s, a “beep” sound indicated the beginning of the haptic exploration phase, during which subjects haptically explored the size of the cylindrical object placed beyond the panel. The haptic exploration phase lasted 2 s, after which a second “beep” sound cued participants to return the hand to the baseline position. After a delay of 2 s, a “go” cue indicated the beginning of the grasping phase, during which subject performed a reach-to-grasp action towards the object that was haptically explored moments earlier. Participants were instructed to grasp the object—without lifting it—from the top using all their fingers, and maintain the grip on the object until they heard a “back” sound 2 s later, which prompted them to return the hand to the baseline position (Fig. 1C).

In summary, we had a 3 × 3 factorial design, with factors SIZE (3 levels: small, medium, large) and GAZE (3 levels: left, centre, right), which led to nine trial types: small object—left fixation, small object—central fixation, small object—right fixation, medium object—left fixation, medium object—centre fixation, medium object—right fixation, large object—left fixation, large object—central fixation, and large object—right fixation. Each trial type was presented 20 times in randomized order; therefore, there were 180 trials in total, separated into 10 blocks.

Before starting the experiment, participants performed 3 training trials to get familiar with the task. In addition, participants underwent a preliminary procedure to validate the coordinates of eye position for each fixation cross for off-line investigations of subjects’ performance during the experiment. To this aim, participants were instructed to fixate each of the three crosses for 4 s in random order. This procedure was repeated 5 times, leading to 15 fixation trials.

### Control experiment

We used the same set-up for a control experiment aimed to determine whether a putative effect of gaze direction on fingers’ movements occurs irrespectively of whether the action is object directed or not. Hence, we asked our participants to perform intransitive opening and closing movements while fixating central and peripheral fixations. The intransitive movement consisted of opening and closing the hand above the table. The apparatus used for the control experiment was identical to the experimental session, with the exception that the cylindrical objects were not used in the control task. As shown in Fig. 1D, at the beginning of each trial participants fixated the cross instructed by the recorded voice (“blue” for left, “red” for centre, “yellow” for right). After 3 s, a “beep” sound instructed participants to move the right hand forward, below the black panel, and open it approximately 7 cm above the table until a second “beep” occurred 3 s later and prompted the subjects to return the hand to the baseline position. The experimental design consisted of 3 levels of the factor GAZE (left, centre, right). Each trial type was presented 20 times, for a total of 60 trials presented in 5 blocks. For each block, trials were presented in random order and each condition was repeated 4 times. Before starting the control experiment, participants completed 3 training trials to get familiar with the task and underwent the preliminary procedure to validate the coordinates of eye position for each fixation cross.

### Grip aperture measurement and eye movements

**Figure 2:**
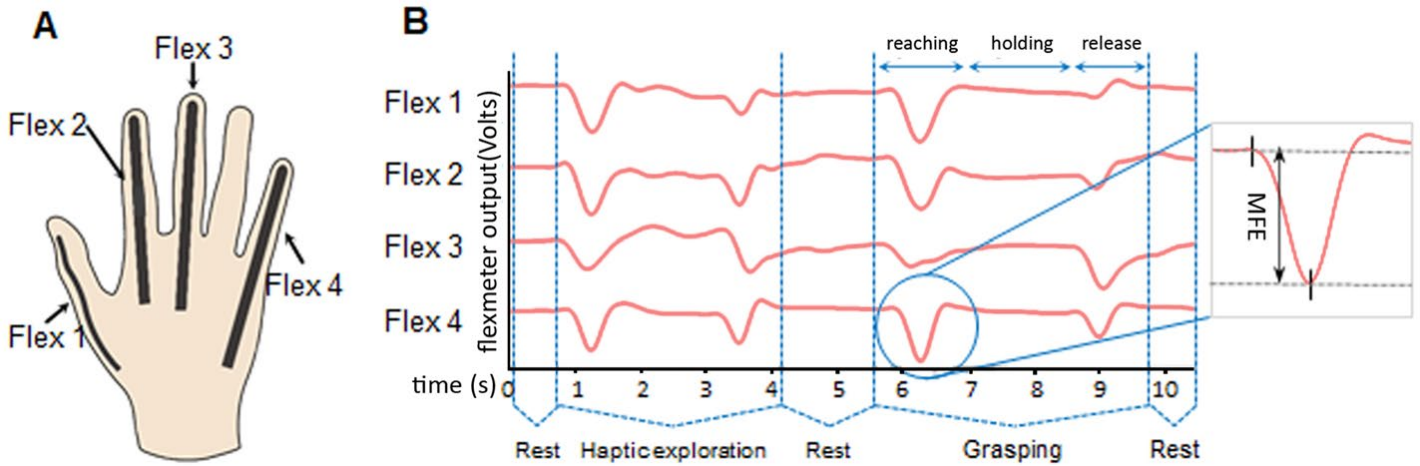
Glove used for acquisition of hand configuration and relative data. (**A**) Schema of the position of flexible sensors of the hand. Participants wore a glove equipped with flexible sensors that allowed us to measure MFE. There was a sensor for the thumb (flex 1), two for the index (flex 2 and 3), one for the middle finger (flex 4), and one for the little finger (flex 5). Each sensor consisted of a resistance variable to its own flexion. The sensors were connected to an analogical-digital converter so that we could quantify the voltage variations of each resistor. (**B**) Signal generated by the voltage variations of each sensor for a whole trial recorded with Signal™ software. For each trial, the pattern was clear and consistent enough to allow us to recognize the phases of the ongoing trial (rest—haptic exploration—rest—grasping) and, more specifically, the reach-to-grasp action: outgoing phase, contact with the object (hold), return phase (release). In the reaching phase, MFE was calculated as the absolute value of the difference between the voltage recorded in the baseline position and the peak during the outgoing phase of the grasping movement, immediately before participants’ fingers held the cylinder. The Figure was processed by means of the GIMP (Gnu Image Manipulation Program) software.

To measure the maximum grip aperture of the participants’ right hand we calculated the *maximum finger extension* (MFE) of all digits during the reach-to-grasp movement. This gave us an index of the hand shaping during the reaching. The literature on visually-guided grasp tells us that the peak aperture is already fully influenced by, and therefore informative of, the target size^30^. Similarly to previous investigation on fingers dynamics^37–40^, we measured MFE, by means of a custom-made glove made of a stretchable fabric of Lycra equipped with four flexible resistive bend sensors strategically placed to detect the voltage deriving from the extension of each finger involved in whole-hand grasps (Fig. 2A). Specifically, we measured the signal from 4 114-mm-long flexion sensors (flexsensors 4.5’’ – Spectrasymbol, USA) placed as follows: one on the thumb (flex 1), one on the index finger (flex 2), one on the middle finger (flex 3) and the last one on the little finger (flex 4). We did not measure the flexion of the ring finger because its movements are strongly correlated with those of the little finger regardless of the goal of the grasp^41–43^. The information on ring finger movements is therefore largely redundant compared to the little finger. The glove was connected to a computer through an analog–digital converter (1401 Power CED) and data was acquired with the Signal™ software, which allowed us to obtain a pattern of sensors’ flexion. The output of the sensors was sampled at 100 Hz rate intervals. The frames recorded in Signal for each trial generated a pattern that allowed us to accurately recognize each phases of both the whole trial (rest – haptic exploration – rest – grasping) and, more importantly, the reach-to-grasp action: outgoing phase, contact with the object (hold), return phase (release) (Fig. 2B). The MFE was calculated as the absolute value of the difference between the voltage recorded during the baseline position and the peak during the outgoing phase of the grasping movement, before the participant’s fingers touched the object. To make the glove suitable and comfortable for each participant, we had it in three different sizes and used the most appropriate one for each subject, depending on the hand size. We recorded the participants gaze coordinates using a Gazepoint GP3 eye-tracker (mean distance from nasion ± SD in exp 1: 67.55 ± 1.82). We used the software OpenSesame^44^ for the stimuli presentation, eye-tracker and fingers movements recording, as well as to trigger the CED for the registration of fingers’ movements from glove’s sensors with Signal software.

### Data analysis

For each participant, the ocular coordinates for each trial were compared with the ones recorded during the preliminary phase of fixation validation, in order to determine whether the fixation was in the correct spatial location, in both the exploration and the movement phases. We excluded trials in which participants moved their gaze away from the fixation point during the trial allowing a fixation of window of 3° of visual angles (eye movements > 3° of visual angles in horizontal and vertical dimension). We discarded 3% of total trials for fixation errors. To make the data from each finger comparable, we normalized the MFEs within each finger and within each participant by means of z-score normalization. For each experimental condition, we removed trials whose MFE deviated more than 2 standard deviations from the mean of each glove’s sensor (4.4% in the main experiment, 4.1% in control experiment). In addition, we excluded trials whose kinematics pattern recorded by Signal™ did not allow for a clear identification of the MFE (1% in the main experiment, 1.2% in control experiment).

We hypothesized that if the perceptual representation of a haptically-explored object is influenced by gaze direction, there would be a change of MFE when participants grasped each of the three sized objects while fixating peripheral directions (left and right) as compared to central fixation (corresponding to the occluded object location). In addition, we also examined MFE’s standard deviation, as uncertainty about the target object might also be reflected in the variability of grip aperture besides than an increase in its mean value.

To test our hypothesis, we performed a repeated measures ANOVA with 3 within-subjects factors: FINGER (4 levels: thumb, index, middle finger, little finger) × GAZE (3 levels: Left, Centre, Right) × SIZE (3 levels: Small, Medium, Large). To have a comprehensive view of the kinematics of the whole hand during reach-to-grasp actions towards haptically explored objects, we analysed data from all fingers involved in the precision whole-hand grasps. For the control experiment, we conducted a repeated measures ANOVA with 2 within-subjects factors: FINGER (4 levels: thumb, index, middle finger, little finger) × GAZE (3 levels: Left, Centre, Right) to assess whether gaze direction influences hand shaping during non-object directed movements. Significance threshold was set at 0.05. Where interactions reached significance, we performed post-hoc tests with Tukey’s Honestly Significant Difference (HSD) test. Effect sizes of significant results were reported as partial eta-squared (p-eta^2^) coefficients.

## Results

### Main experiment: FINGER × GAZE × SIZE ANOVA

The ANOVA with 3 within-subjects factors (FINGER × GAZE × SIZE) conducted on the average MFE in the main experiment revealed a main effect of GAZE (F(2,28) = 4.98; *p* = 0.01; p-eta^2^ = 0.26), and a main effect of SIZE (F(2,28) = 47.77; *p* < 0.0000001; p-eta^2^ = 0.77). In addition, we observed significant FINGER x SIZE (F(2,28) = 4.46; *p* < 0.0006; p-eta^2^ = 0.24) and FINGER x GAZE (F(2,28) = 3.22; *p* = 0.002; p-eta^2^ = 0.22) interactions. Nonsignificant results included the main effect of FINGER (F(3,42) = 1.16; *p* = 0.34; p-eta^2^ = 0.07), the GAZE x SIZE interaction (F(2,28) = 0.60; *p* = 0.66; p-eta^2^ = 0.04) and the FINGER × GAZE × SIZE interaction (F(12,168) = 0.86; *p* = 0.58; p-eta^2^ = 0.06). All results are given in Table 1.

**Table 1:** z-score transformed Maximal Finger Extension (MFE) in all experimental conditions of the main experiment. Mean values (Standard Error) are given.

Finger	Thumb			Index			Middle			Little		
Object	Small	Medium	Large	Small	Medium	Large	Small	Medium	Large	Small	Medium	Large
Left gaze	− 0.22 (0.14)	0.05 (0.07)	0.22 (0.16)	− 0.22 (0.09)	− 0.07 (0.05)	0.37 (0.08)	− 0.54 (0.09)	− 0.03 (0.06)	0.33 (0.05)	− 0.64 (0.07)	− 0.11 (0.05)	0.54 (0.09)
Central gaze	− 0.2 (0.12)	− 0.12 (0.05)	0.3 (0.15)	− 0.33 (0.09)	− 0.05 (0.05)	0.3 (0.07)	− 0.59 (0.08)	0.09 (0.05)	0.42 (0.08)	− 0.65 (0.07)	− 0.04 (0.05)	0.56 (0.07)
Right gaze	− 0.25 (0.11)	− 0.05 (0.07)	0.23 (0.14)	− 0.31 (0.09)	0.0 (0.04)	0.3 (0.07)	− 0.44 (0.09)	0.18 (0.06)	0.48 (0.07)	− 0.48 (0.09)	0.11 (0.06)	0.69 (0.09)

### Main experiment: FINGER × SIZE interaction

**Figure 3:**
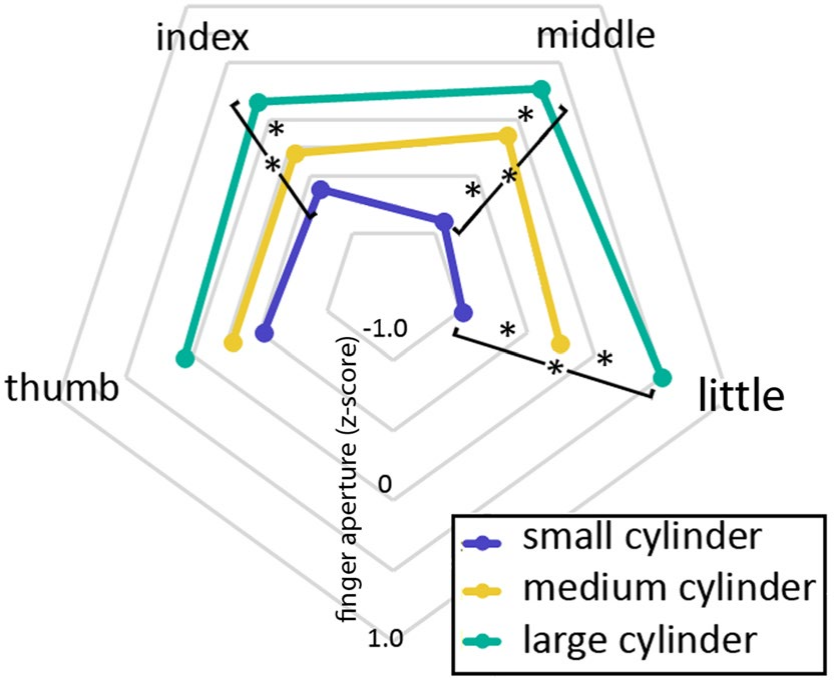
Mean z-score transformed maximum finger extension (MFEs). The diagram shows the individual finger excursions according to object size in the main experiment. The Figure was processed by means of the GIMP (Gnu Image Manipulation Program) software.

A significant FINGER × SIZE (F(2,28) = 4.46; *p* < 0.0006; p-eta^2^ = 0.24) was found, illustrated in Fig. 3. We further investigated such interaction were by means of four separate 1-way ANOVAs, one for each of the four fingers. The 1-way ANOVA on the thumb data did not show any main effect of SIZE (F(2, 28) = 3.13, *p* = 0.059). The 1-way ANOVA on the index finger data showed a main effect of SIZE (F(2, 28) = 17.44, *p* = 0.00001). Post-hoc analyses with Tukey’s Honestly Significant Test showed that MFEs to the small object were not different from those to the medium object (*p* = 0.06), but they were significantly different from those to the large object (*p* = 0.0001) and that MFEs to the medium object were significantly different from those to the large object (*p* = 0.004). The 1-way ANOVA on the middle finger data showed a main effect of SIZE (F(2, 28) = 55.22, *p* < 0.000001). Post-hoc analyses with Tukey’s HSD showed that MFEs to the small object were different from those to the medium object (*p* = 0.0001) and from those to the large object (*p* = 0.0001) and that MFEs to the medium object were significantly different from those to the large object (*p* = 0.003). The 1-way ANOVA on the little finger data showed a main effect of SIZE (F(2, 28) = 65.13, *p* < 0.000001). Post-hoc analyses with Tukey’s HSD showed that MFEs to the small object were different from those to the medium object (*p* = 0.0001) and from those to the large object (*p* = 0.0001) and that MFEs to the medium object were significantly different from those to the large object (*p* = 0.001). To summarize, the FINGER x SIZE interaction implied that not all fingers scaled accurately to object size. In fact, we found that there was a gradient of fingers mobility from the thumb to the little finger. The thumb’s MFE failed to show significant modulation from object size. The index finger showed a weak and incomplete correlation with object size, while the middle and little fingers showed a clear linear correlation with object size, which was even more robust for the little finger than for the middle finger. This result confirms the accurate measurement of our glove and demonstrates that MFE scales with object size during memory-based grasping actions towards tactile stimuli. The mobility gradient observed here, from thumb to little finger is difficult to relate to the previous literature because the vast majority of studies investigating the effect of object size on grip aperture describe only the thumb-index pair, ignoring the remaining fingers. Thus, we cannot be sure whether this pattern is specific to our recording apparatus, specific to our task or, possibly a general pattern.

### Main experiment: FINGER × GAZE interaction

**Figure 4:**
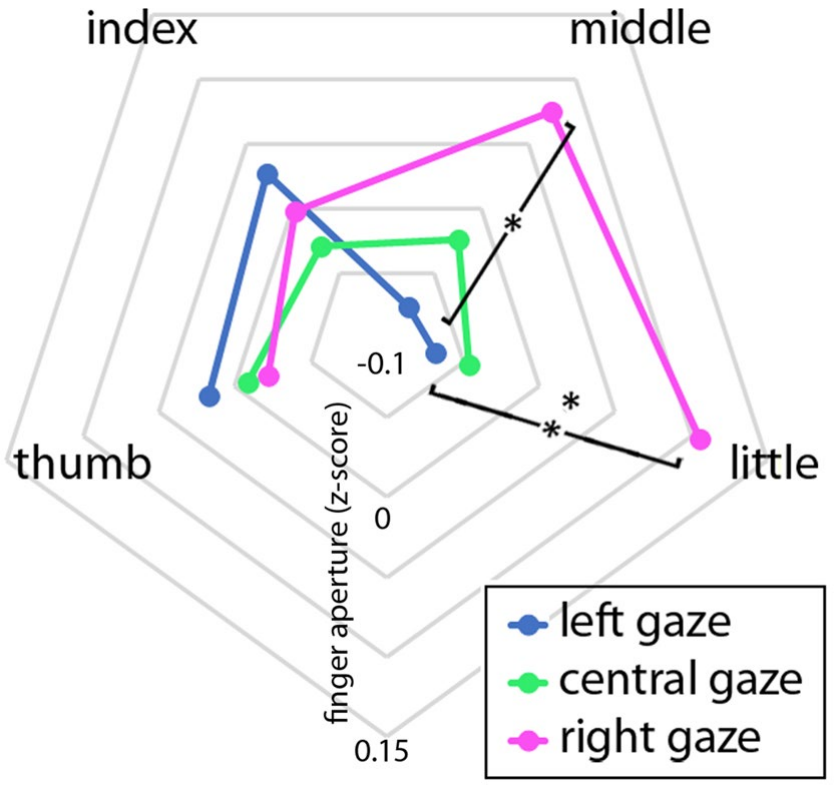
Mean z-score transformed maximum finger extension (MFEs). The diagram shows the individual finger excursions according to gaze direction in the main experiment. The Figure was processed by means of the GIMP (Gnu Image Manipulation Program) software.

We observed a significant FINGER × GAZE (F(2,28) = 3.22; *p* = 0.002; p-eta^2^ = 0.22) interaction, that is illustrated in Fig. 4. The 2-way interaction was further investigated by means of four separate 1-way ANOVAs, one for each of the four fingers. No significant effect of GAZE was found in the ANOVAs on the thumb (F(2, 28) = 0.44, *p* = 0. 65) and index (F(2, 28) = 0.97, *p* = 0.39) MFEs. The ANOVA on the middle finger showed a significant main effect of GAZE (F(2, 28) = 4.37, *p* = 0.022). Post hoc tests using Tukey’s HSD showed that MFEs with left gaze were significantly smaller than MFEs with right gaze (*p* = 0.018). MFEs in central position of gaze were not significantly different from the left and right gaze conditions. The ANOVA on the little finger showed a significant main effect of GAZE (F(2, 28) = 11.79, *p* = 0.00002. Post hoc tests using Tukey’s HSD showed that MFEs with left gaze were significantly smaller than MFEs with right gaze (*p* = 0.0004). MSFEs in central position of gaze were significantly different from the right gaze condition (*p* = 0.0017), but not from MFEs in left gaze.

### Main experiment: analysis on MFE variability

In addition to analysing the MFE, we also explored whether eccentricity could affect hand shaping in terms of variability of fingers’ movement, as uncertainty about object size might increase when participants fixate away from the object. Hence, we analysed the standard deviation of MFE by means of an ANOVA with 3 within-subjects factors (FINGER x GAZE x SIZE) that showed a main effect of SIZE (F(2,28) = 7.49; *p* = 0.003; p-eta^2^ = 0.19 ) with higher variability of MFE for the Large vs. Small (*p* < 0.001) and Large vs. Medium object (*p* = 0.02). Since no other significant effect or interaction was observed (all *p* > 0.16), this analysis showed no effect of eccentricity on grip aperture variability.

### Control experiment

The FINGER x GAZE 2 within-subject factors ANOVA on MFEs of participants performing intransitive (non-object directed) movements failed to show any effect. The main effect of FINGER (F(3, 42) = 1.20, *p* = 0.32), the main effect of GAZE (F(2, 28) = 1.21, *p* = 0.31) and the FINGER x GAZE interactions show non-significant results. We concluded that gaze direction did not affect fingers’ movements during intransitive actions. The mean MFEs are shown in Table 2.

**Table 2:** z-score transformed Maximal Finger Apertures (MFEs) in all experimental conditions of the control experiment. Mean values (Standard Error) are given.

Finger	Thumb	Index	Middle	Little
Left gaze	− 0.03 (0.04)	− 0.07 (0.03)	− 0.01 (0.03)	− 0.03 (0.06)
Central gaze	0.03 (0.03)	0.09 (0.03)	0.03 (0.03)	− 0.01 (0.05)
Right gaze	0.0 (0.05)	− 0.02 (0.04)	− 0.02 (0.04)	0.03 (0.06)

## Discussion

### Main findings

The present results indicate that finger aperture, while grasping towards haptically explored objects are significantly modulated by gaze direction. A control experiment in which participants performed intransitive, non-object-directed hand movements failed to show any effect of gaze direction. Similar evidence showing gaze-dependency of reaching movements towards proprioceptive targets has been previously shown for spatial targets in blind and sighted individuals^32^. Here for the first time we show an effect of gaze in the dimension of grasping. Object properties (such as size) that are perceived with touch are mapped in coordinates that are dependent of gaze direction.

The modulation was evident in the middle and, to a greater extent, in the little finger. The thumb and index finger failed to show any modulation from gaze. In both the index and middle fingers, the finger aperture was larger when the participant was looking right, and smaller when the participant was looking left. It is interesting to note that the thumb in the present data failed to show modulation even by object size (Fig. 3) and similarly, the index finger appeared to be less mobile than the last two fingers. Grip aperture is expected to scale with object size. Indeed, we found a robust effect of object size on the average finger aperture, but deeper analysis showed a gradient of motility of the fingers, from the thumb, which stayed almost fixed, to the most mobile little finger. Such gradient in movement range and in adaptation to object size could explain why we found effects of gaze only in the middle and little fingers, i.e. in the ones that showed higher mobility and task-related flexibility.

In the control experiment we showed that in the absence of a target object, gaze direction failed to modulate hand shaping. The value of this control experiments is to be evaluated according to a subtraction logic: the features present in the control task include the gaze manipulation and the finger movement. The main task also contains the elements of haptic acquisition of the object geometry, its storage in memory and its recall. We can therefore capitalize on the null results of the experiment by hypothesizing that the effects of gaze are not systematically present whenever a movement is made away from fixation points, but rather are specific to transitive, goal-directed movements. In the absence of more control conditions we cannot state that the effects of gaze are exclusive to the task of keeping in memory and using a haptically-acquired object image.

The aim of the experiment was to find configuration errors (i.e. errors due to a reference frame shift) in haptically-guided grasping, when manipulating the eye-centred frame of reference. The main results here confirm that shifts in eye position produce configuration errors when grasping an unseen object, supposedly associated with gaze direction.

### Comparison with the effects of gaze direction on visually guided grasping

One possible explanation of the present data is comparing them to the effects of gaze direction on visually guided grasping. Behavioural studies have shown that several aspects of grasping movements display more conservative grasping behaviours when there is higher visual uncertainty about the target object, and the uncertainty about an object’s properties increases when the object is in peripheral vision^45–48^. In particular, during grasping actions, uncertainty about the target object is reflected in wider grip apertures during the movement, as this behaviour avoids collisions with the object^49^. Besides the effects of sensory uncertainty, it is possible that effects of eccentricity of vision are due to the fact that the central (foveal) and peripheral visual field may be supported by separated neural systems as shown by dissociations between central and peripheral grasping in neuropsychological research. Eccentric grasping is typically impaired in optic ataxia^50^ but also in putative ventral stream lesions as shown by Hesse et al.^51^.

In line with this, investigations about the kinematics of grasping movements have consistently shown wider grip apertures during actions directed at objects that are away from our gaze as opposed to within our central visual field^29,36,52^. Evidence about the influence of gaze direction on grasping behaviour has been replicated in several visual conditions, including continuous visual information about the target and hand throughout the movement^29,53^ as well as during no online visual information about target and hand during the movement. In addition, similar results have been found when the location of the gaze varied with respect to a fixed target location, as well as when the location of the target varied with respect to a fixed gaze direction, suggesting that the observed effects are indeed related to the increasing distance of the target relative to fixation rather than the body. Further, different studies have shown similar effects independent of whether participants had their head fixed or not, indicating that the effect is independent of the direction of the head^29,36,52^. Summing up, during vision, the effect of gaze is symmetrical and depends on eccentricity of the target. In the present data we failed to show such a pattern, and on the contrary, we found an asymmetrical effect, with larger apertures when looking right compared to when looking left or centrally. The finding is fully justified by the fact that precision requirements are actually the same, and  the explanation related to eccentricity and uncertainty does not fit he present data.

It is possible that the asymmetry observed in our results could be related to the use of the participants’ right hand. However, previous studies showed no difference between left and right hand when comparing the kinematics of both hands for grasping in conditions of occluded vision^54,55^. While it is likely that the combination of the hand used and the visual field in which the action was performed caused the observed asymmetry (despite the lack of visual information about the object) further investigations are required to disambiguate this issue.

### Dependency of haptically-acquired object geometry from eye position indirectly supports the EVC’s role as a memory sketchpad buffer in the early visual cortex

Recent evidence shows that the EVC in sighted individuals is also involved in tactile perception of objects despite the lack of visual information^7–9,12,56–58^ as well as during action execution towards haptically explored objects^59^. In addition, the activity pattern in the EVC allows decoding action planning seconds before participants perform a movement towards a visible target, and this cannot be entirely explained by motor imagery^60^. These findings suggest that the EVC is involved in mechanisms that go well beyond visual processing, and in line with this, a theory describing the EVC as an all-purpose cognitive and spatial blackboard has been proposed^61^. Thus, it would seem possible that haptically explored objects as well as subsequent action plans are represented and stored in eye-centred coordinates with similar mechanisms as engaged for visually explored objects and visually guided actions. Our finding showing effects of eye position on haptic grasping indicate that haptic memory for objects could rely at some point on an eye-centred frame of reference. This finding fits extremely well with the idea that visual system activity in non-visual behaviour is causally related to performance. However, this effect of gaze direction needs to be further examined in future investigations in which other frames of reference (i.e. head- or body-centred) will be considered.

### Limitations

The present study has several limitations. First, here we kept object position constant and manipulated eye position. A full factorial design, testing also eccentric object position, shifts in head or body-centred frames of reference, and the left hand could provide more complete information. Second, while we hypothesize that the observed effects are a distortion of the memory of haptically-obtained object geometry, we cannot clearly describe the way the object’s representation changes. Testing different object shapes and different orientations could help in characterizing this feature. In a related topic, the lack of effects of gaze on the index and thumb pinch could be hypothetically explained by the fact that their action during ecological grip is mainly oriented along a vertical axis, i.e. orthogonal to the direction of gaze. The little finger on the contrary, acts on a predominantly horizontal axis, parallel to the changes in gaze. It is possible that changes in object memory induced by gaze changes follow the horizontal direction. Third, it is the possible that effects of gaze direction on grip aperture may be related to eccentricity of the peripheral gaze directions (about 15° of visual angle). Further studies should investigate different and larger eccentricities (up to ~ 50° as in other studies^36,52^). Finally, to investigate into the asymmetry of the effect of gaze observed here, further investigations are needed in which participants use the right or the left hand in the same task, and in which participants grasp objects with vision, using the same manipulations. This would allow the deflections and asymmetry (which would presumably replicate in the new experiment) to be interpreted more adequately as departures of one extent or another from typical performance.
